# The Presence of Clitoromegaly in the Nonclassical Form of 21-Hydroxylase Deficiency Could Be Partially Modulated by the CAG Polymorphic Tract of the Androgen Receptor Gene

**DOI:** 10.1371/journal.pone.0148548

**Published:** 2016-02-05

**Authors:** Vivian Oliveira Moura-Massari, Flávia Siqueira Cunha, Larissa Garcia Gomes, Diogo Bugano Diniz Gomes, José Antônio Miguel Marcondes, Guiomar Madureira, Berenice Bilharinho de Mendonca, Tânia A. Sartori Sanchez Bachega

**Affiliations:** 1 Unidade de Suprarrenal, Laboratório de Hormônios e Genética Molecular LIM 42, Disciplina de Endocrinologia, Hospital das Clínicas, Faculdade de Medicina da Universidade de São Paulo, Sao Paulo, Brazil; 2 Departamento de Clínica Médica, Hospital das Clínicas, Faculdade de Medicina da Universidade de São Paulo, Sao Paulo, Brazil; University Hospital S. Maria della Misericordia, Udine, ITALY

## Abstract

**Background:**

In the nonclassical form (NC), good correlation has been observed between genotypes and 17OH-progesterone (17-OHP) levels. However, this correlation was not identified with regard to the severity of hyperandrogenic manifestations, which could depend on interindividual variability in peripheral androgen sensitivity. Androgen action is modulated by the polymorphic CAG tract (nCAG) of the androgen receptor (AR) gene and by polymorphisms in 5α-reductase type 2 (*SRD5A2*) enzyme, both of which are involved in the severity of hyperandrogenic disorders.

**Objectives:**

To analyze whether nCAG-AR and *SRD5A2* polymorphisms influence the severity of the nonclassical phenotype.

**Patients:**

NC patients (n = 114) diagnosed by stimulated-17OHP ≥10 ng/mL were divided into groups according to the beginning of hyperandrogenic manifestations (pediatric and adolescent/adult) and *CYP21A2* genotypes (C/C: homozygosis for mild mutations; A/C: compound heterozygosis for severe/mild mutations).

**Methods:**

*CYP21A2* mutations were screened by allelic-specific PCR, MLPA and/or sequencing. *HpaII*-digested and *HpaII*-undigested DNA samples underwent GeneScan analysis to study nCAG, and the S*RD5A2* polymorphisms were screened by RLFP.

**Results:**

Mean nCAG did not differ among pediatric, adolescent/adult and asymptomatic subjects. In the C/C genotype, we observed a significantly lower frequency of longer CAG alleles in pediatric patients than in adolescent/adults (p = 0.01). In patients carrying the A/C genotype, the frequencies of shorter and longer CAG alleles did not differ between pediatric patients and adolescent/adults (p>0.05). Patients with clitoromegaly had significantly lower weighted CAG biallelic mean than those without it: 19.1±2.7 and 21.6±2.5, respectively (p = 0.007), independent of the *CYP21A2* genotype's severity. The *SRD5A2* polymorphisms were not associated with the variability of hyperandrogenic NC phenotypes.

**Conclusions:**

In this series, we observed a modulatory effect of the CAG-AR tract on clinical manifestations of the NC form. Although the NC form is a monogenic disorder, our preliminary data suggested that the interindividual variability of the hyperandrogenic phenotype could arise from polygenic interactions.

## Introduction

Steroid 21-hydroxylase deficiency is the most frequent cause of congenital adrenal hyperplasia (CAH), accounting for more than 90 to 95% of CAH cases [1, 2]. Due to a lack of negative feedback from cortisol, ACTH stimulation increases, shifting the precursors of steroidogenesis toward androgen synthesis. There is a spectrum of clinical forms, traditionally divided into classical and nonclassical (NC) forms. In the classical form, in addition to the manifestations of cortisol insufficiency, female patients usually present with prenatal external genital virilization, and both sexes present with postnatal virilization. Additionally, approximately 70–80% of patients also present with severe impairment of aldosterone production, resulting in hyponatremic dehydration during the first weeks of life. In the NC form, the hyperandrogenic signs begin later in life: children typically present with precocious pubarche, while adolescents and adults present with hirsutism, menstrual abnormalities and/or infertility. Clitoromegaly has also been observed in both children and adults, occurring in up to 7–10% of NC cases [3]. In fact, these classical and NC forms reflect different impairments of enzymatic activity caused by *CYP21A2* mutations.

In CAH, there is a good correlation between genotypes and phenotypes; that is, homozygous patients carrying mutations resulting in total/severe (<7%) and moderate (20–50%) enzymatic activity impairments generally present with the classical and NC forms, respectively [4–9].

Genotypes predicting the NC form carry mild mutations in homozygosis or in compound heterozygosis with severe mutations. Despite the presence of the mild allele, some studies have reported a modulatory effect of the severe allele on the NC phenotype: these patients presented higher ACTH-stimulated 17OH-progesterone (17OHP) levels, higher basal androgen levels and/or earlier onset of hyperandrogenic manifestations compared to those who are homozygous for mild mutations. [10–13] However, this hypothesis has been debated in the literature because other reports have not found these correlations [14–15]. Previously, in our cohort consisting of 114 NC patients, we did not identify correlation among the severity of NC genotypes, age at the beginning of hyperandrogenic manifestations, basal serum androgen levels and the presence of virilizing signs. Interestingly, similar frequencies of both NC genotypes (mild and severe) were observed in asymptomatic female patients and in those with slight clitoromegaly [16]. These data suggested that individual differences in the peripheral androgen sensitivity, modulated by the androgen receptor and 5-α-reductase type 2 genes, could account for this phenotypic variability.

Androgens act via the androgen receptor (AR), the gene for which carries a polymorphic CAG tract at exon 1, varying from 8 to 35 repeats in the normal population [17, 18]. Variations in the CAG repeat numbers (nCAG) have been inversely correlated with AR transactivation activity and consequently with androgen phenotypic variability. Shorter tracts have been associated with idiopathic precocious pubarche, increased severity and earlier age onset of prostate cancer, whereas longer CAG tracts have been associated with oligospermic infertility [19–22].

Dihydrotestosterone (DHT), the main active androgen binding to the androgen receptor (*AR*), is converted from testosterone by the action of 5α-reductase type 2 enzyme. The 5α-reductase type 2 gene can carry two frequent polymorphisms, V89L and A49T, which modify the enzymatic activity and influence the phenotypic variability of androgen-dependent disorders. The A49T variant might play a role in the etiology and progression of prostate cancer, while the V89L variant has been strongly associated with hypospadias risk in children and also with protection against PCOS [23–26].

Based on these findings, in this study of a noteworthy NC cohort, we evaluated the modulatory effects of androgen receptor and 5α-reductase type 2 gene variants on the hyperandrogenic phenotypic variability of NC patients.

## Materials and Methods

This study protocol was approved by the Ethics Committee of the Hospital das Clínicas, Universidade de São Paulo, and written informed consent was obtained from all of the patients and/or their caretakers.

Most patients were from São Paulo state and presented with late onset hyperandrogenic manifestations and hormonal diagnosis of NC-CAH, which was defined by basal 17OHP levels ≥ 10 ng/mL or by ACTH-stimulated 17OHP ≥ 10 ng/mL at 60 min after i.v. injection of synthetic 1–24 ACTH (0.25 mg) [27, 28]. All of the patients had a defined molecular NC genotype, i.e., mutations identified in both *CYP21A2* alleles, and according to these criteria, 114 patients were selected [16].

Fifty-three patients (45 females) presented at early or middle childhood with precocious pubarche (6 ± 1.9 years old), and 50 patients (all females) presented at adolescence or adulthood with hirsutism, menstrual abnormalities and/or infertility as their chief complaint (23 ± 11.3 years old). The other 11 cases (5 females) were asymptomatic and were diagnosed during familial molecular studies. Slight clitoromegaly was defined by a clitoris length >9 mm in children and >16 mm in adult females, evaluated by a single examiner [29, 30]. Clitoromegaly was observed in 10 of 114 patients (3 children and 7 adults), but separate urethral and vaginal openings were identified in all of these patients. The mean duration of patient follow-up was 11.0 ± 7.5 years.

Clinical and hormonal data from patients were retrospectively obtained from medical records. These data were correlated with nCAG of the *AR* gene and with 5α-reductase type 2 allelic variants, and the following manifestations were analyzed: precocious pubarche, amenorrhea, oligomenorrhea, infertility, hirsutism and clitoromegaly. Precocious pubarche was defined by the appearance of pubic hair before 8 years old in girls and before 9 years old in boys. Amenorrhea was defined by the absence of menstrual periods for at least 3 consecutive months and oligomenorrhea by fewer than 6 menstrual periods in the previous year. Infertility was defined by the inability to conceive within 18 months of unprotected intercourse. Hirsutism was defined by male pattern of body hair distribution and a Ferriman and Galley score (FG) ≥ 8. The severity of hirsutism was classified into 2 groups: mild (≤ 14 FG) and severe (≥ 15 FG) [31–33]. Symptomatic patients were grouped according to the beginning of clinical manifestations into pediatric and adolescent/adult groups. None of the subjects had taken any medication for at least 3 months.

### Hormone Assays

Serum 17OHP levels were measured by radioimmunoassay (Diagnostic System Laboratories INC/USA, Webster, TX, USA). Cortisol, progesterone and testosterone levels were measured by immunofluorometric assays (AutoDELFIA, Wallac, Finland). Androstenedione levels were determined by chemiluminescence assay (Immulite 2000, Siemens Health Care, UK). The intra- and inter-assay coefficients of variation varied from 5% to 10% [16].

### Molecular Studies

*CYP21A2* (Gene ID 201910) point mutations were screened using allelic-specific PCR and/or *CYP21A2* sequencing, including of the promoter and intronic regions [10, 34, 35]. Large gene rearrangements (*CYP21A2* deletions and large gene conversions) were screened by Southern blotting and/or MLPA techniques (SALSA P50B CAH MLPA Mix, MRC-Holland BV, Amsterdam, the Netherlands) [33].

#### Patient groups

Patient genotypes were classified according to the predicted impairment in enzymatic activity observed in the *in vitro* studies of 3 groups: A/C (severe), B/C (moderate) and C/C (mild) nonclassical genotypes. Basically, A alleles carry mutations predicting total or almost total impairment of enzymatic activity: *CYP21A2* deletion, large gene conversion, IV2-2A>G, p.G110Efs, exon 6 cluster (p.I236N, p.V237E, p.M239K), p.Leu307fs, p.R356W, p.Q318X, p.G424S, p.Arg483fs or the IVS2-13 A/C>G (I2 splice) mutations. B alleles carry the p.I172N mutation, resulting in 3 ± 7% residual enzymatic activity, and C alleles carry the p.P30L, p.V281L or p.P453S mutations, resulting in 20 ± 60% residual enzymatic activity [7, 8, 36, 37].

#### X-chromosome inactivation analysis

Methylation of the *Hpa*II site, close to the CAG repeats, is correlated with X-inactivation. This site is methylated in the inactive X chromosome, and it resists to cleavage by the *Hpa*II enzyme; therefore, a PCR product is obtained only from the inactive X-chromosome. For each DNA sample, two reactions were performed; in one, 2μg of DNA was digested with 20 U of *Hpa*II (CCGG) at 37°C overnight. A second reaction was similar to the previous one, except for the absence of the enzyme. The reactions were stopped by incubation at 96°C for 5 min. Both digested and undigested DNA (100 ng) were used for PCR amplifications of the CAG polymorphic tract of the *AR* gene, as previously described [38, 39]. PCR products were submitted to capillary electrophoresis on an ABI PRISM 310 Genetic Analyzer (Applied Biosystems, Foster City, CA USA) and were analyzed by GeneScan software to determine the sizes of the amplified fragments, which were established from their comparisons with size markers submitted to electrophoresis in the same run. These sizes were correlated with the CAG repeat numbers [18]. The relative inactivation pattern of the AR alleles was calculated using the correction described by Pegoraro et al. [40]. The area of the allele with a smaller repeat number (which is preferentially amplified) was divided by the area of the allele with the greatest number of repeats to determine how many times the first was preferentially amplified. After digestion with *HpaII*, this factor was applied to the area of the larger allele to normalize the level of amplification between the two alleles. We considered the amplification of one allele ≥ 75% as skewed X-chromosome inactivation [38, 41, 42]. The results of X-inactivation analysis were used to generate a mean value that represented differences in the expression of constituent alleles. It was achieved by multiplying each allele in a genotypic pair by its percentage of total expression (100 minus % inactivity) and by totaling the two adjusted repeat values to achieve a new mean value, which we called the X-weighted biallelic mean [43].

#### S*RD5A2* polymorphisms

Screening for the V89L and A49T SRD5A2 allelic variants was performed using 3 UI of the restriction enzymes *RsaI* (GT//CA) and *MwoI* (GCNNNNN//NNGC) (New England Biolabs, Beverly, MA, USA), respectively, and 10 μL of the PCR product of exon 1 at 37°C overnight in a final reaction volume of 20 μL [44]. The restriction products were analyzed by electrophoresis on 3% agarose gel. To monitor the size of the fragments generated by restriction enzymes, the molecular weight marker ΦX174/ Hae*III* was used (Invitrogen, Life Technology, Gaithersburg, MD, USA). Each reaction was performed with a known control sample, without polymorphisms.

### Statistical Analysis

Qualitative variables were compared using the chi-squared test. Quantitative data, after normality testing, were compared using Wilcoxon’s test or Student’s *t*-test.

For the multivariate analysis, modification of the linear regression, which considered different patterns of genetic inheritance (recessive, dominant or codominant), was used [45]. Traditional linear regression, adjusted for covariates of interest, was performed and three results were provided based on recessive inheritance (recessive, dominant or codominant). For the linear regression, we included hormonal data, clinical data, *CYP21A2* genotype, mean CAG repeat number and *SRD5A2* alleles. For univariate statistical analysis, p<0.05 was considered significant, and 95% confidence intervals were generated that did not include the unit. For multivariate analyzes, we planned Bonferroni’s correction for multiple comparisons; however, because no associations attained statistical significance, this correction was not necessary. All of the statistical analyses were performed using Small Stata software, version 11.1 (2010 StataCorp., College Station, TX, USA).

## Results

The patients were divided into three groups according to their ages at the beginning of manifestations: pediatric (n = 53), adolescent/adult ≥ 12 years old (n = 50), and asymptomatic (n = 11).

The CAG repeat numbers presented a normal distribution, and 88% of women were heterozygous for this number. The mean CAG repeat numbers in pediatric, adolescent/adult and asymptomatic groups were 22.2 ± 3.1 (12–30), 22.6 ± 3.5 (16–31) and 22.4 ± 3.6 (14–28), respectively (p>0.05). The allelic frequency of CAG repeats was also evaluated among these three groups. The frequencies of shorter CAG alleles (≤ 18 repeats) in the pediatric, adolescent/adult and asymptomatic groups were 15%, 14% and 9%, respectively (p>0.05). The frequencies of longer CAG alleles (≥ 26 repeats) were 6.6% in the pediatric group, 16% in the adolescent/adult group and 13% in the asymptomatic group (p> 0.05).

Nonclassical genotypes from the A/C (severe) and C/C (mild) groups were identified in 52% and 48% of patients, respectively. Only one patient carried the B/C genotype group, and this patient was excluded from the analysis. Initially, frequencies of CAG alleles were compared among the patient groups carrying the same *CYP21A2* genotype group. Among the patients carrying the A/C *CYP21A2* genotype, frequencies of shorter and longer CAG alleles did not differ between the pediatric and adolescent/adult groups (p>0.05). Among patients carrying the C/C *CYP21A2* genotype, the frequency of longer CAG alleles was significantly greater in adolescent/adult than in the pediatric group (p = 0.01), whereas the frequencies of shorter alleles did not differ between these groups (p>0.05) (Table 1). The CAG distribution and period of manifestations according to most frequent *CYP21A2* genotypes were described in Table 2.

**Table 1:** Frequencies of shorter (≤ 18 repeats) and longer (≥ 26 repeats) CAG alleles between pediatric and adolescent/ adult groups according to *CYP21A2* genotypes.

	A/C genotype group			C/C genotype group		
	Age group	*CYP21A2* genotype	Allelic frequency (%)	Age group	*CYP21A2* genotype	Allelic frequency (%)
**Longer alleles**	Pediatric	A/C	13	Pediatric	C/C	16
	Adolescent/ adult	A/C	18 p = 0.5	Adolescent/ adult	C/C	9 p = 0.28
**Shorter alleles**	Pediatric	A/C	10	Pediatric	C/C	2
	Adolescent/ adult	A/C	15 p = 0.37	Adolescent/ adult	C/C	17 p = 0.01

**Table 2:** Most frequent *CYP21A2* genotypes, CAG allelic distribution and period of onset of hyperandrogenic manifestations.

*CYP21A2* genotype	Patients n (%)	Mean nCAG	Shorter Alleles n (%)	Period of manifestations	
				childhood (n)	adult (n)
V281L/V281L	42 (36.8)	21.3 ± 2.8	9 (10.7)	16	26
I2Sp/V281L	15 (13.2)	21.3 ± 3.2	4 (13.3)	8	7
LR/V281L	12 (10.5)	22.6 ± 1.2	0	4	8
P453S/V281L	7 (6.1)	21 ± 3.6	3 (21.4)	4	3
R356W/V281L	4 (3.5)	22.7 ± 1.3	0	3	1
Del 8nt/V281L	3 (2.6)	19.6 ± 2.1	2 (33.3)	2	1

LR: large gene rearrangements, included large gene conversions and the *CYP21A2* deletions; Del: deletion

Subsequently, the frequencies of shorter and longer CAG alleles were compared between *CYP21A2* genotypes in patients from the same group according to the beginning of manifestations. The frequencies of longer CAG alleles between pediatric patients carrying the A/C and C/C *CYP21A2* genotypes were 10% and 2%, respectively (p = 0.09); no difference was observed in the frequency of shorter CAG alleles. Regarding adolescent/adult patients carrying the A/C and C/C genotypes, no difference was observed in the frequencies of shorter and longer alleles (Table 1).

Skewed X-chromosome inactivation was observed in 13% of NC females, and its frequency did not differ in female subjects between the pediatric and adolescent/adult groups (23% *vs* 10%, respectively) (p>0.05).

The weighted biallelic CAG mean was also evaluated according to the number of hyperandrogenic symptoms at diagnosis. In asymptomatic patients, in patients with one symptom and in those with two or more symptoms, the mean CAGs were 21.8 ± 2, 21.2 ± 2.9 and 21.6 ± 2.8, respectively (p>0.05). In the hirsute patients, the weighted biallelic CAG mean was 20.9 ± 2.5, and in those without hirsutism it was 21.7 ± 2.8 (p> 0.05). Ferriman scores were compared across 33 women who were first evaluated before the use of any topical and/or antiandrogen therapies. In patients with severe hirsutism (Ferriman score ≥ 15), the weighted biallelic CAG mean was 21.5 ± 3.0 and, in those with mild hirsutism (Ferriman score ≤ 14), it was 19.9 ± 2.3 (p>0.05).

**Fig 1:**
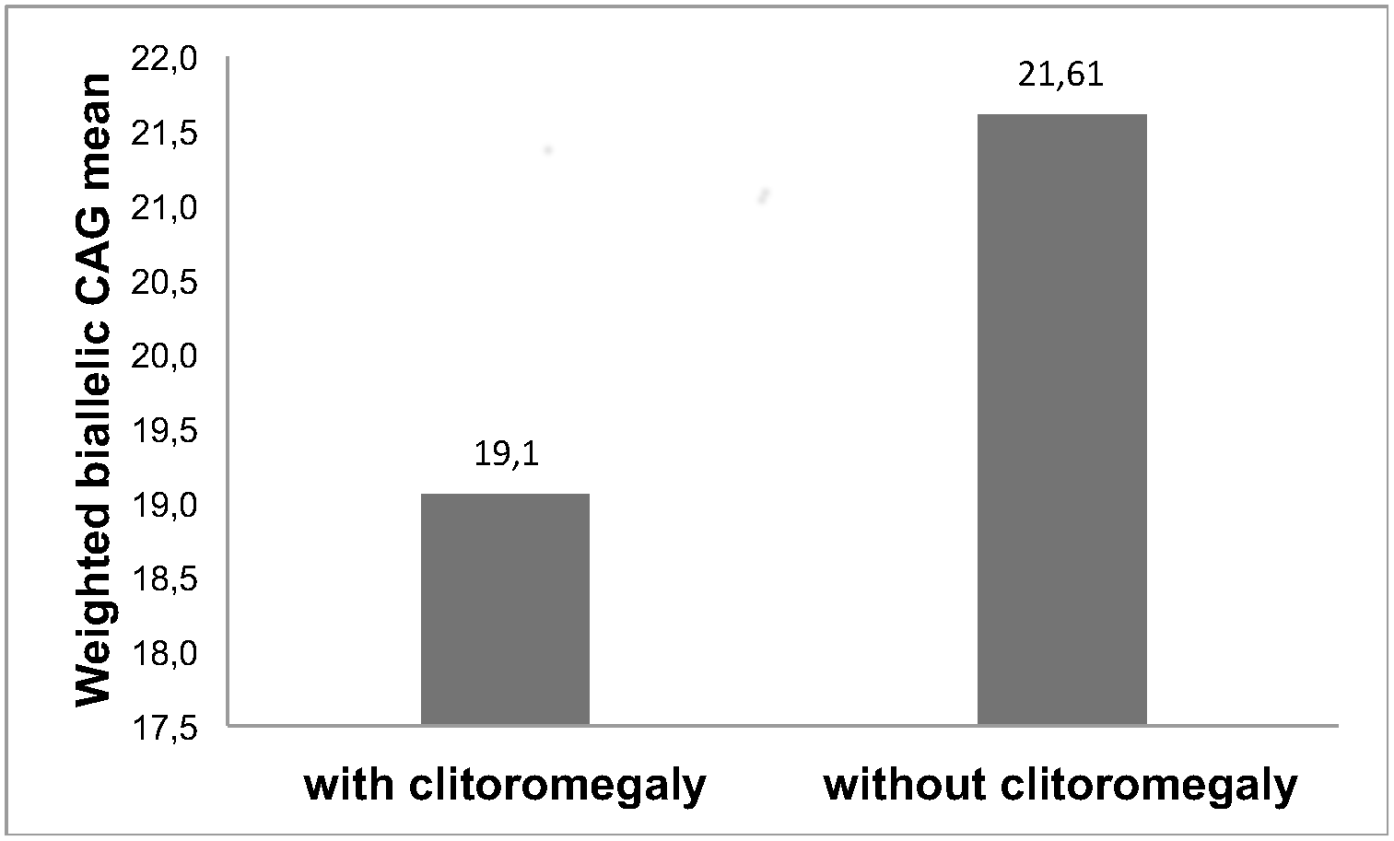
Weighted biallelic CAG mean of the AR gene in NC-CAH patients with and without clitoromegaly.

The influence of CAG repeats on the presence of virilization was also evaluated and the weighted biallelic CAG mean was compared between patients´ groups with and without clitoromegaly. The weighted biallelic CAG mean was significantly lower in patients with clitoromegaly than in those without it: 19.1 ± 2.7 *vs* 21.6 ± 2.5, respectively (p = 0.007) (Fig 1). Eight of ten (80%) patients with clitoromegaly and 14 of 93 (15%) without clitoromegaly carried shorter CAG alleles (≤ 18 repeats) (p<0.001). There were no differences in serum testosterone levels between adult women with and without clitoromegaly: 138 ± 73 ng/dL and 94 ± 66 ng/dL, respectively (p>0.05).

The V89L-*SRD5A2* variant was identified in 31% of alleles in Hardy-Weinberg equilibrium; 45% of the patients were heterozygous, and 9% were homozygous carriers. Unlike the V89L, the A49T variant was rare in this series (1% of alleles) and was excluded from association analysis. No difference in the frequency of the V89L variant was observed regarding the onset of symptoms, severity of hirsutism and presence of clitoromegaly in patients carrying both *CYP21A2* genotypes.

## Discussion

The NC form presents great phenotypic variability, even among subjects carrying similar genotypes. Additionally, in the literature, it has not been well defined whether the severe allele, in a compound heterozygous patient, modulates the severity of hyperandrogenic manifestations. Previous studies have reported conflicting results regarding the association between the severity of the NC genotype and the onset of symptoms [12–15]. In this context, we emphasized two studies with large NC cohorts that found no influence of *CYP21A2* genotypes on the onset and/or severity of hyperandrogenic manifestations [11, 16].

The phenotypic variability of hyperandrogenism in the NC form could result from interindividual differences in peripheral androgen sensitivity, which might arise from genetic variants related to androgen action and/or metabolism.

The influence of these variants has been widely evaluated in hyperandrogenic disorders. Some studies have identified that shorter CAG alleles of the AR gene present with greater frequency in women with PCOS in relation to the normal population [46, 47]. However, other studies did not find this association [48, 49], and it is likely that these discordant findings result from methodological differences, such as those for defining the criteria for X-inactivation skewing and for defining shorter CAG alleles. An influence of the CAG tract was observed on the phenotypic severity of prostate cancer, and a significant correlation between shorter alleles and an earlier age at diagnosis was identified [50, 51].

In this present study, besides the great miscegenation of Brazilian population, we found a normal distribution of CAG repeat numbers in the NC cohort, and no differences were observed compared to our normal population or with Caucasians [18, 52]. It seems more important to evaluate the influence of the CAG tract at the beginning of manifestations in the NC form; we hypothesized that pediatric group would carry a higher frequency of shorter CAG alleles, since they present at younger age. However, the weighted biallelic CAG mean, which reflects both the number of repeats and the percentage of activity of each allele, did not differ between NC patients from the pediatric and adolescent/adult groups. This weighted biallelic mean did not differ, even when we considered the severity of the 21-hydroxylase genotype. We cannot exclude that these negative results could be related to a sample size effect. It is worth emphasizing the higher frequency of longer CAG tracts in the adult group bearing the mild NC genotype compared with the pediatric group bearing the same genotype; consequently, we speculated that this higher frequency of alleles with lower AR activity contributed to the later onset of symptoms in patients carrying similar genotypes.

In the same manner, the influence of CAG repeat numbers was previously analyzed at the beginning and with regard to the severity of hyperandrogenic manifestations in the NC form. Ben-Shachar et al. [53], analyzing a group of 119 NC female patients, identified that patients carrying shorter CAG alleles (< 25 repeats) had a higher frequency of precocious pubarche and precocious puberty. Ibáñez et al. [54], in a cohort of 181 women, found that shorter CAG alleles (≤ 20 repeats) were associated with increased risk for premature pubarche relative to the normal population and also with subsequent development of ovarian hyperandrogenism. Interestingly, Hickey et al. [43] found that shorter CAG alleles (≤ 22 repeats) were associated with infertility in a subset of 122 PCOS patients. In contrast, our study revealed no influence of nCAG on the prevalence of premature pubarche, hirsutism, menstrual abnormality or infertility or in the number of manifestations at diagnosis, such as isolated hirsutism and/or associated with menstrual irregularity. Similarly to our results, it was observed in a large PCOS cohort that CAG repeats and serum testosterone levels were not predict factors of hirsutism and acne [49]. However, we would like to emphasize that our criteria for classifying shorter alleles (≤ 18 repeats) might have been stricter than those used in the studies cited above.

To assess the severity of hyperandrogenism, we used the Ferriman score for hirsutism and the presence of an enlarged clitoris, which indicates a stronger phenotype of hyperandrogenism. Association analyses disclosed no influence of the weighted biallelic CAG mean on the severity of hirsutism, similar to the findings of Dasgupta et al. [48], who evaluated PCOS patients. However, the number of hirsute NC patients, who started follow-up in our service without ever having received treatment before, was small. Furthermore, we also speculated that small variations in the CAG repeats within the normal range might not be significant in the modulation of hirsutism. Although this is a large cohort considering nonclassical patients, probably to definitively exclude the influence of CAG repeats in the less severe hyperandrogenic manifestations, besides clitoromegaly, it should be necessary higher number of patients.

Interestingly, in our patients with clitoromegaly, basal androgen concentrations were not different from those in subjects without clitoromegaly [49], but we noted a positive association between shorter CAG alleles and the presence of clitoromegaly, as well as with a lower weighted biallelic CAG mean. This finding reinforced those observed in a previous study conducted in our laboratory in patients with the classical form of CAH, in whom we found an influence of the CAG tract in the phenotypic variability of external genitalia virilization [18]. In men, the influence of the CAG tract on the phenotype of the external genitalia was also reported. Ogata et al. [55] described a patient with 46,XY and with genital ambiguity, carrying a rare allele with 44 CAG repeats, without any other allelic variant in exonic/intronic regions of the *AR* gene and no changes in testosterone synthesis.

Interestingly, no differences in CAG allelic distributions were observed between our asymptomatic and symptomatic NC female subjects. Asymptomatic NC females despite the 21-hydroxylase deficiency, presented with normal basal androgen levels through consecutive evaluations. It is likely that this cryptic phenotype is related to genetic variations in androgen synthesis. However, in this study few asymptomatic cases were evaluated, since they were diagnosed during familial study of an index case.

We also call attention to the process of gene regulation of the androgen receptor, which is extremely complex and differs in various tissues according to the activity of co-regulatory proteins. These proteins might also play a modulatory role in peripheral sensitivity to androgens and corroborate the phenotypic variability of the NC form [56].

Another protein of importance in the peripheral action of androgens is the 5α-reductase enzyme. The peripheral action of testosterone is boosted by the activity of this enzyme converting testosterone into dihydrotestosterone, which is a more potent androgen than testosterone, largely responsible for the growth and stimulation of hair follicles. The increased peripheral activity of 5α-reductase was considered a predisposing factor for the development of idiopathic hirsutism and precocious idiopathic pubarche [57].

Allelic variants in the *SRD5A2* gene have also been associated with the development of androgen-dependent disorders, such as prostate cancer and PCOS. In a series enrolling 187 PCOS women, the status carrier for the 89L allele, which is known to reduce the activity of 5α-reductase type 2, was associated with a lower likelihood of developing PCOS [23]. However, this variant was not correlated with a lower intensity of hirsutism. The 89L variant was identified among approximately 31% of the alleles in our patients, similar to the normal Brazilian population [58]. In contrast, our association studies have not identified an influence of the 89L allele on the hyperandrogenic phenotype of the NC form. This allele was not protective against the development of premature pubarche, and its frequency did not differ significantly between hirsute and non-hirsute patients and or between those with and without menstrual irregularity.

Our NC patients carrying the 89L variant had lower median scores for hirsutism and later onset of manifestations relative to wild-type carriers, but these differences were not significant. Although this variant decreased the activity of 5α-reductase to 30% *in vitro* [59], it is possible that was not significant *in vivo* for the NC phenotype. It is likely that the 5α-reductase type 1 enzyme is more important to the severity of hirsutism, as previously reported in women in PCOS [23].

In conclusion, we described that the CAG tract of the androgen receptor gene could explain, at least partially, the phenotypic variability in the NC form, but these data must be replicated in other populations. Although the NC form of CAH is a monogenic disorder, our preliminary data suggested that the interindividual variability in the hyperandrogenic phenotype could arise from polygenic interactions.

## Supporting Information

S1 Fig Weighted biallelic CAG mean of the AR gene in NC-CAH patients with and without clitoromegaly. (TIF)

S1 Table Frequencies of shorter (≤ 18 repeats) and longer (≥ 26 repeats) CAG alleles between pediatric and adolescent/ adult groups according to *CYP21A2* genotypes. (PDF)

S2 Table Most frequent *CYP21A2* genotypes, CAG allelic distribution and period of onset of hyperandrogenic manifestations. LR: large gene rearrangements, included large gene conversions and the *CYP21A2* deletions; Del: deletion.
(PDF)
