# Supplementary figures and images for: The Presence of Clitoromegaly in the Nonclassical Form of 21-Hydroxylase Deficiency Could Be Partially Modulated by the CAG Polymorphic Tract of the Androgen Receptor Gene
Source: PLoS One. 2016 Feb 5;11(2):e0148548. doi: 10.1371/journal.pone.0148548 (PMC4744051; doi:10.1371/journal.pone.0148548)

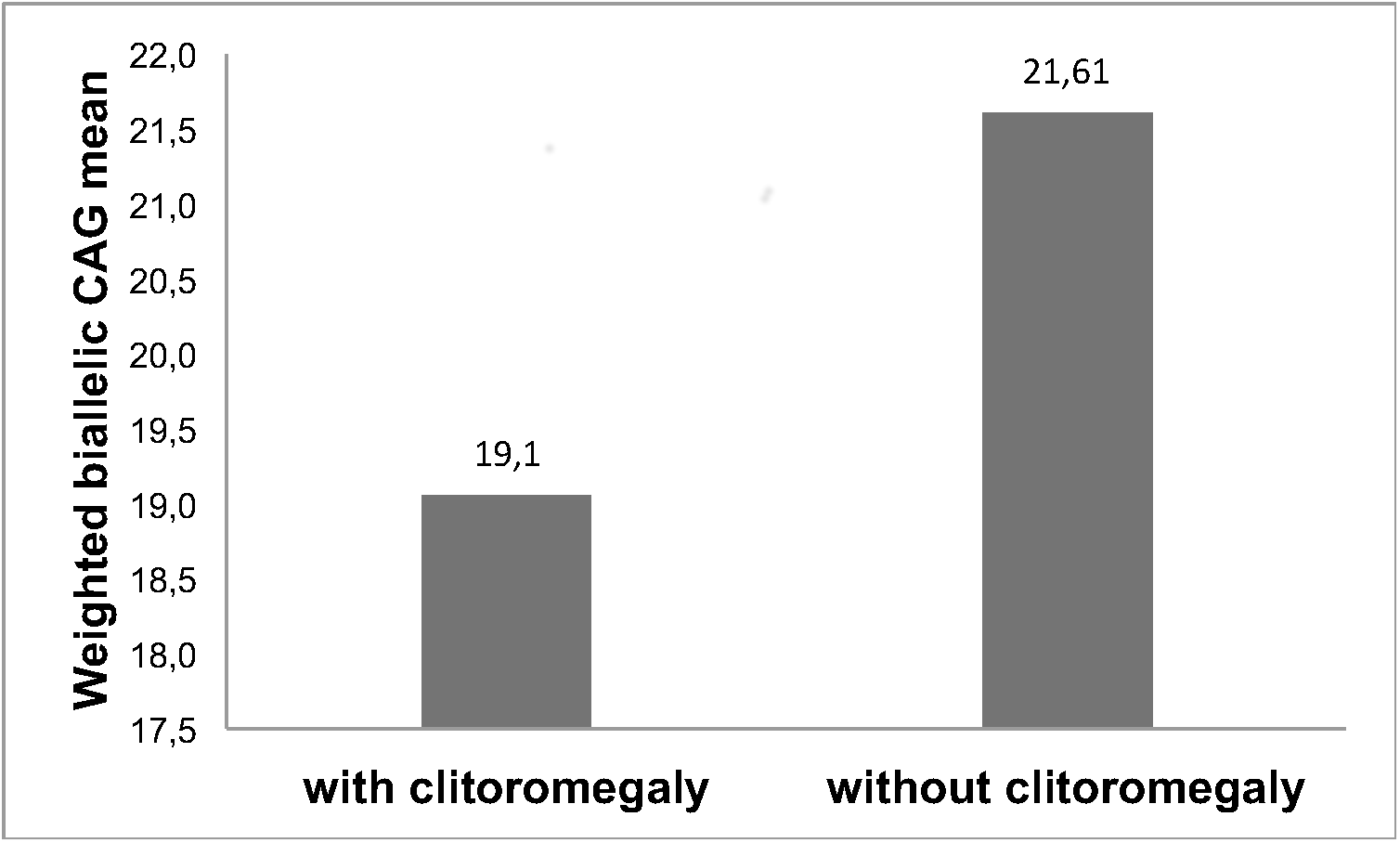

Supplement: S1 Fig — (TIF) [file pone.0148548.s001.tif]
