# Supplementary material for: The Presence of Clitoromegaly in the Nonclassical Form of 21-Hydroxylase Deficiency Could Be Partially Modulated by the CAG Polymorphic Tract of the Androgen Receptor Gene
Source: PLoS One. 2016 Feb 5;11(2):e0148548. doi: 10.1371/journal.pone.0148548 (PMC4744051; doi:10.1371/journal.pone.0148548)
Supplement: S1 Table — (PDF) [file pone.0148548.s002.pdf]

	A/C genotype group			C/C genotype group		
	Age group	CYP21A2 genotype	Allelic frequency (%)	Age group	CYP21A2 genotype	Allelic frequency (%)
Longer alleles	Pediatric	A/C	13	Pediatric	C/C	16
	Adolescent/ adult	A/C	18<br>p=0.5	Adolescent/ adult	C/C	9<br>p=0.28
Shorter alleles	Pediatric	A/C	10	Pediatric	C/C	2
	Adolescent/ adult	A/C	15<br>p=0.37	Adolescent/ adult	C/C	17<br>p=0.01
