# Supplementary material for: The Presence of Clitoromegaly in the Nonclassical Form of 21-Hydroxylase Deficiency Could Be Partially Modulated by the CAG Polymorphic Tract of the Androgen Receptor Gene
Source: PLoS One. 2016 Feb 5;11(2):e0148548. doi: 10.1371/journal.pone.0148548 (PMC4744051; doi:10.1371/journal.pone.0148548)
Supplement: S2 Table — LR: large gene rearrangements, included large gene conversions and the CYP21A2 deletions; Del: deletion. (PDF) [file pone.0148548.s003.pdf]

CYP21A2 genotype	Patients n (%)	Mean nCAG	Shorter Alleles n (%)	Period of manifestations	
				childhood (n)	adult (n)
V281L/V281L	42 (36.8)	21.3 ± 2.8	9 (10.7)	16	26
I2Sp/V281L	15 (13.2)	21.3 ± 3.2	4 (13.3)	8	7
LR/V281L	12 (10.5)	22.6 ± 1.2	0	4	8
P453S/V281L	7 (6.1)	21 ± 3.6	3 (21.4)	4	3
R356W/V281L	4 (3.5)	22.7 ± 1.3	0	3	1
Del 8nt/V281L	3 (2.6)	19.6 ± 2.1	2 (33.3)	2	1
